# Localization of Liv2 as an Immature Hepatocyte Marker in EB Outgrowth

**DOI:** 10.1100/tsw.2009.18

**Published:** 2009-03-01

**Authors:** Ikkei Takashimizu, Yoshiki Tanaka, Susumu Yoshie, Yoshiya Kano, Hinako Ichikawa, Li Cui, Naoko Ogiwara, Kohei Johkura, Katsunori Sasaki

**Affiliations:** Department of Anatomy and Organ Technology, School of Medicine, Shinshu University, Matsumoto, Japan

**Keywords:** mouse liver, development, mouse ES cells, immature hepatocytes, hepatoblasts, EB outgrowth, Liv2, immunohistochemistry, TEM

## Abstract

The objective of this study was to establish Liv2, a surface marker of mouse immature hepatocytes (hepatoblasts), as a selection tool for embryonic stem (ES) cell–derived immature hepatocytes by acquiring basic data on Liv2 in normal mouse embryos and by confirming Liv2 expression in mouse ES-derived cells. The estimated molecular weight of Liv2 was 40–45 kDa, and immunoreactivity was definitively detected in the cell membrane of fetal hepatocytes on embryonic day (E) 9.5, declined gradually until E12.5, and subsequently became undetectable. Liv2 was localized on and close to the cell membrane. Embryoid bodies (EB) were formed from mouse ES cells whose undifferentiated state was confirmed with immunostaining of Nanog by the hanging drop method. A few Liv2-positive cells occurred as a cluster in EB outgrowth on day 7, but only some of these were albumin (ALB)-positive on day 13. These cells had the same pattern of immunoreactivity, i.e., localization on the cell membrane, as immature hepatocytes in the developing liver, although there were other types of cells with a different pattern of immunoreactivity that were seen only as a granular pattern in the cytoplasm and without ALB or the neuronal marker nestin. These results suggest that Liv2 may be useful as a surface marker for immature hepatocytes derived from ES cells. This application would allow for the sole selection of immature hepatocytes and provide a useful tool for regenerative medicine.

## INTRODUCTION

Organ transplantation is a procedure marked by a severe lack of donors; however, embryonic stem (ES) cells are a promising alternative form of therapy with two major benefits. The first is the multipotency of ES cells[2,10], which makes it possible to produce any type of tissue from a single resource[3,5,8,9,

15,16,17,18]. The second is that ES cells are an infinite resource because they are able to grow continuously in the undifferentiated state under appropriate conditions.

ES cells differentiate through embryoid bodies (EB), which are similar to embryos at the egg-cylinder stage[1], into multiple cell types, making it necessary to discriminate and isolate them to obtain specific cells. Therefore, it is necessary to discriminate and isolate wanted cells from unwanted ones. To date, various diffusible cytoplasmic markers, such as albumin (ALB), alpha-fetoprotein (AFP), and glucose-6-phosphatase (G6P), have been reported as useful for identifying immature and mature hepatocytes; however, these are not surface markers, so it is impossible to isolate only living hepatocytes using fluorescence-activated cell sorting (FACS) or magnetic-activated cell sorting (MACS) methods. An isolating method using several surface antigens of hematopoietic markers to select hepatocytes has been reported[12], but the method is insufficient due to inaccurate cell counts and contamination of other types of cells in addition to hematopoietic cells and hepatocytes.

Liv2, which was first reported by Watanabe et al.[14], may be an optimal marker for selecting ES cell–derived immature hepatocytes. This antigen is selectively detected in the fetal liver and has two features: it is recognized in the fetal hepatocyte, i.e., hepatoblasts (described as immature hepatocytes in this paper), and it decreases from embryonic day (E) 9.5 to E12[4,14]. Liv2 is restricted to the cell membrane, but not to the cytoplasm or nuclei[11,14], so it can be used as a surface marker to select immature hepatocytes. Selecting immature hepatocytes, due to their high proliferation rate, is more convenient than selecting mature hepatocytes for transplantation; however, there is little known about molecular weight and fate during development. Further, to select immature hepatocytes by FACS or MACS, it has to be confirmed definitely that Liv2 is a surface antigen; however, its microlocalization has not yet been investigated.

In this study, we used western blotting, immunohistochemistry, transmission electron microscopy (TEM), and immuno-TEM (ImmunoGold) to gather basic information about Liv2 and to determine its utility as a marker to detect immature hepatocytes derived from ES cells.

## MATERIALS AND METHODS

### ES Cells and Cell Cultures

The karyotypically normal ES cell line, ES-D3, which was developed by Doetschman et al.[1], was obtained from the American Type Culture Collection (Manassas, VA) and used in this study. Frozen stocks of cells were thawed, seeded onto feeder cell layers, and cultured as described by other investigators[5]. Briefly, ES cells were cultured in Dulbecco’s modified Eagle’s medium (DMEM; GIBCO, Grand Island, NY) supplemented with 15% lot-tested fetal bovine serum (FBS), 100 μ*M* nonessential amino acids (GIBCO), 1 m*M* sodium pyruvate (GIBCO), 100 μ*M* 2-mercaptoethanol (Sigma, St. Louis, MO), and 1000 U/ml of leukemia inhibitory factor (LIF; Chemicon, Temecula, CA) on a feeder cell layer of mitomycin C–inactivated mouse embryonic fibroblasts under standard culture conditions (5% $CO_2$ and humidified air 37°C). The cells were subcultured every 2 days to maintain an undifferentiated phenotype.

### EB Formation

The cells were dissociated with 0.1% trypsin-EDTA and resuspended in Iscove’s modified Dulbecco’s medium (IMDM; GIBCO) containing 20% FBS, 100 μ*M* nonessential amino acids, 1 m*M* sodium pyruvate, and 100 μ*M* 2-mercaptoethanol without LIF at a concentration of 1000 cells/50 μl drop. The cells were cultured in 5% $CO_2$ at 37°C for 5 days using the hanging drop method, and EBs that formed in the drops were replated onto gelatin-coated 24-well plates.

## SDS-PAGE and Western Blot

Livers of E11.5 embryos were used as anti-Liv2 antibody-positive controls. Livers removed from E11.5 embryos under a stereoscopic microscope were sonicated directly in SDS reducing buffer (BIO-RAD, Hercules, CA) and boiled for 4 min following the manufacturer's recommendations.

## Immunocytochemistry

After rinsing with phosphate-buffered saline (PBS) at 37°C, the EB outgrowths at various stages were fixed in 4% paraformaldehyde/0.1 *M* phosphate buffer (pH 7.4) for 20 min at room temperature. They were then rinsed three times with 20 m*M* PBS (pH 7.4). Prior to immunostaining, the specimens were treated in 0.2% Triton X-100/20 m*M* PBS (pH 7.4) for 10 min for permeabilization and rinsed three times in 20 m*M* PBS (pH 7.4). The specimens were singly or doubly stained with specific antibodies for Nanog as an undifferentiated cell marker (rabbit polyclonal; ReproCELL, Tokyo, Japan), Liv2 as a marker of immature hepatocytes (rat monoclonal; MBL International, Woburn, MA), ALB as a marker of mature hepatocytes (rabbit polyclonal; DAKO, Carpinteria, CA), and nestin as a marker of neurons and ectodermal-originating cells (mouse monoclonal; Chemicon). The specimens were incubated with the appropriate secondary antibody conjugated with AlexaFluor 488 or 568 (Molecular Probes, Eugene, OR), nucleus counterstained with 4',6-diamidino-2-phenylindole, dihydrochloride (DAPI; Molecular Probes), and observed with a Leica LSM TCS SP2 AOBS CLSM equipped with Ar, He/Ne, and diode lasers (Leica Microsystems GmbH, Wetzlar, Germany).

## Immunohistochemistry

Livers of mice at each developmental stage (E9.5, E12.5, E14.5, neonatal, and adult) were fixed in 4% paraformaldehyde/0.1 *M* phosphate buffer (pH 7.4) overnight at 4°C, embedded in paraffin, and 5-µm thick serial sections were cut. After deparaffinization and rehydration, the sections were stained with anti-Liv2 antibody. Only the E9.5 livers were subjected to the avidin-biotin-peroxidase complex method followed by diaminobenzidine. Normal goat serum was used instead of the primary antibody for a negative control.

After labeling with appropriate fluorescent secondary antibodies and nuclear counterstaining with DAPI, the sections were observed with a Leica microscope as described above.

## Conventional TEM

TEM was performed as described by other investigators[6]. Briefly, livers of E11.5 embryos and EB outgrowths were fixed in 2.5% glutaraldehyde/45 m*M* cacodylate HCl (pH 7.2) overnight at 4°C. After rinsing three times in 180 m*M* sucrose/80 m*M* cacodylate HCl (pH 7.2) at 4°C for 3 h, the specimens were postfixed in 1% osmium tetroxide/0.1 *M* sodium cacodylate buffer (pH 7.2) for 60 min at 4°C, dehydrated in a graded series of ethanol and embedded in epoxy resin. Resin sections of 1 µm in thickness were stained with toluidine blue solution for light microscopy. Ultrathin sections of the specimens were stained with uranyl acetate and lead citrate.

## Immuno-TEM

Pre- and postembedding methods were performed to determine the microlocalization of Liv2. Livers of E11.5 embryos and EB outgrowths were fixed in 4% paraformaldehyde/0.1% glutaraldehyde/0.1 *M*

phosphate buffer (pH 7.4) at 4°C for 1 h and embedded in Lowicryl K4M at −20°C[7]. Ultrathin sections were immunostained with anti-Liv2 antibody and a protein-G gold solution containing 15-nm gold particles (ICN Biomedicals, Aurora, OH).

Following the pre-embedding method, which is an adequate method to confirm that Liv2 is a definite surface antigen, fetal livers (E11.5) were dissociated with 0.1% trypsin-EDTA and immunoreacted with anti-Liv2 rat IgG (1:100; MBL)/BSA (0.5% normal donkey serum; CALBIOCEM) for 30 min at 4°C. After washing twice, the dissociated cells were reacted with mouse antibiotin iron beads (1:5 dilution; Milltenyi Biotec GmbH, Germany) for 30 min at 4°C. As these iron beads are usually used for MACS, if detected on the membrane of fetal hepatocytes, immature hepatocytes can be selected by MACS. After washing, the samples were fixed with 2% glutaraldehyde overnight and prepared according to a conventional method.

After staining the sections with uranyl acetate or lead citrate, they were observed with a JEOL JEM-1200 electron microscope at an acceleration voltage of 80 kV.

## RESULTS

### Liv2 Specificity

Western blotting analysis was performed to examine Liv2 specificity in E11.5 embryonic livers (Fig. 1a). Only one signal was found at 40–45 kDa, which corresponded to the estimated molecular weight. Based on their molecular weights, the smeared signals above and below the target signal may have been heavy and light mouse IgG chains. The immunoreactivity of Liv2 antibody was confirmed by the ABC method (Fig. 1b,c).

Immunohistochemical analysis was performed using paraffin sections of the liver at E9.5, E12.5, E14.5, neonatal, and adult stages to investigate the localization, developmental fate, and occurrence of Liv2 (Figs. 2 and 3). Liv2 immunoreactivity was most intense in E9.5 livers (Fig. 2a–d) and decreased gradually with development (Fig. 2e–h). No immunoreactivity was observed at the neonatal and adult stages (Fig. 3a–c). Liv2 was restricted to the cell membrane and to small granules close to the cell membrane (Fig. 2c,d), which was confirmed by immuno-TEM (Fig. 4). ImmunoGold particles and iron beads were localized at the cell membrane of the immature hepatocytes (Fig. 4b,c), and gold particles were found in granules under the cell membrane (Fig. 4c).

### *Immature Hepatocytes in EB Outgrowth*

Next, we investigated whether Liv2 recognized only in immature hepatocytes was effective as a marker of immature hepatocytes derived from ES cells. Anti-Nanog antibody was used to confirm that the cells were undifferentiated ES cells. Nanog is a homeodomain protein expressed in the nucleus specifically of multipotent stem cells, such as ES cells, EG cells, and early embryos[14]. Undifferentiated, NANOG-positive ES cells were not immunoreactive for Liv2 (Fig. 5a–c).

EBs are often formed to differentiate ES cells because they mimic early embryo EB outgrowths differentiated spontaneously into ectoderm, mesoderm, and endoderm, and we previously confirmed that they differentiate into hepatocytes[8]. Two days after replating the EBs onto gelatin-coated plates (EB outgrowth), Liv2-positive cells and immature hepatocytes (hepatoblasts) were found as clusters in EB outgrowths (Fig. 5d–g). Similar to the E9.5 immature hepatocytes, there was immunoreactivity localized at the cell membrane and in granules close to the membrane (Fig. 5f,g), but at this stage, there was no immunoreactivity for the hepatocyte marker ALB. However, 13 days after replating the EBs, an immunocytochemical analysis confirmed ALB expression in Liv2-positive clusters (Fig. 5h).

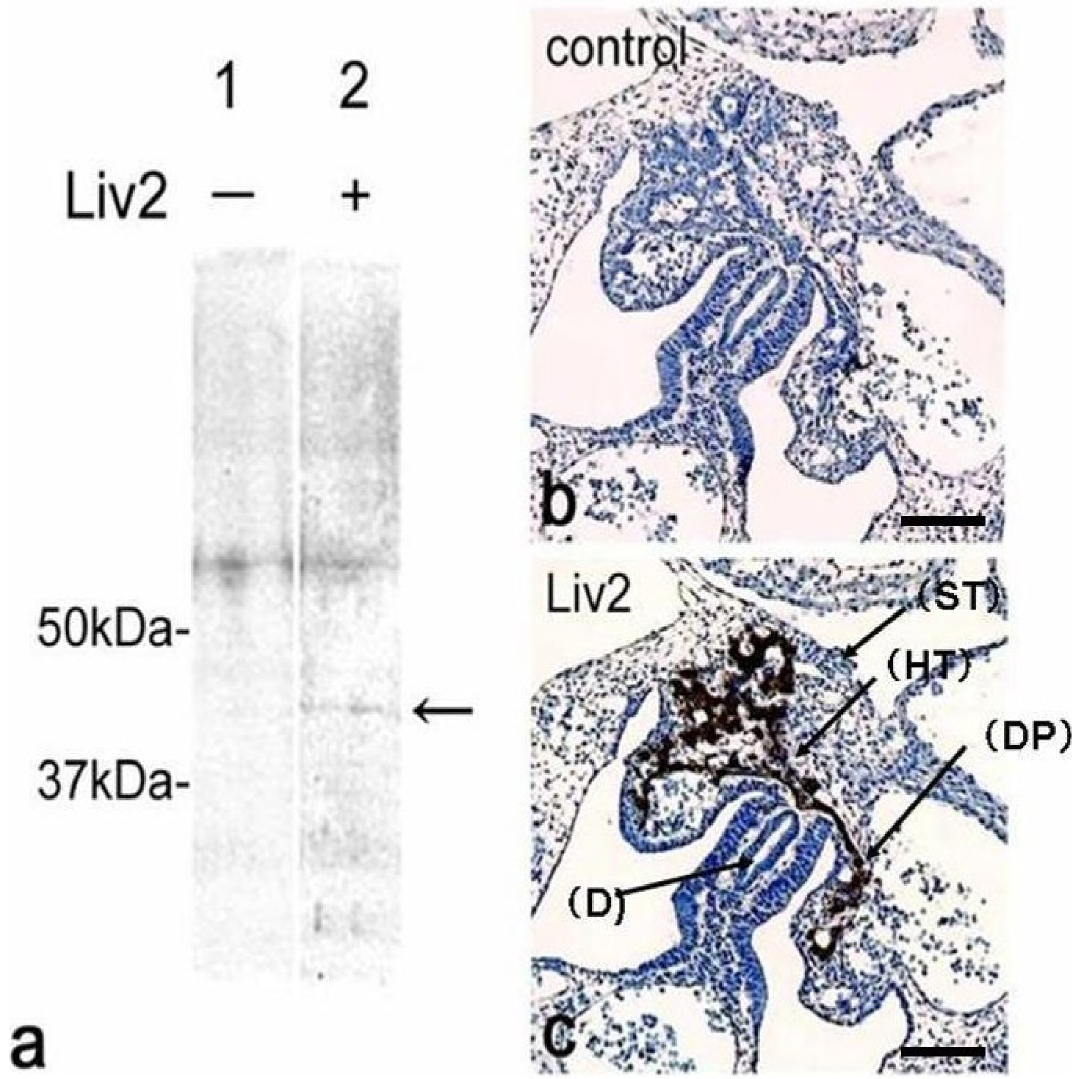

**FIGURE 1.** (a) Specificity of Liv2. Western blotting analysis of Liv2 using E11.5 embryonic liver. Lane 1 was negative control, omitting the step of primary antibody, and lane 2 was positive control. The target signal was observed at the estimated molecular weight of 40–45 kDa (lane 2, arrow). The signals of >50 kDa and <37 kDa were mouse IgG heavy chain and light chain, respectively. Immunohistochemical analysis of E9.5 embryo (b and c) was a section of E9.5 embryo omitting the step of primary antibody. Only immature hepatocytes were immunoreactive for Liv2 (c). D, duodenum; DP, dorsal pancreas; HT, hepatic trabeculae; ST, septum transversum. Bars = 100 µm.

## *Liv2 Granular Immunoreactivity in EBs and Their Characteristics*

In addition to immature hepatocytes, we found another type of Liv2-positive cell with a different immunoreactivity profile. These cells were neither localized at the cell membrane nor in the granules near the cell membrane. They were diffused in a granular pattern in the cytoplasm, but only around the nuclei (Fig. 6a). There were many granules in the cells located in peripheral regions of the EB outgrowth, and their number decreased with time in cell culture (Fig. 6b,c). These granules were not immunoreactive for the hepatocyte marker ALB (Fig. 6d–f). Based on their location in the EB outgrowth, we assumed that these cells may be neurons; however, they were not immunoreactive for the neuronal marker nestin. (Fig. 6g–i) Therefore, although these cells remain to be identified, they were confirmed to be neither hepatocytes nor neurons.

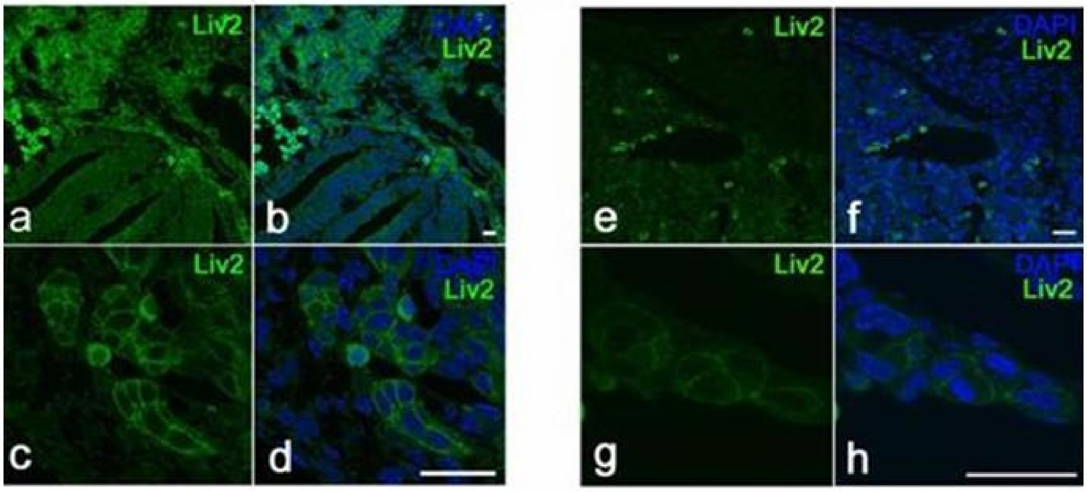

**FIGURE 2**. Immunoreactivity for Liv2 (green) was observed in immature hepatocytes at stages from E9.5 (a–d) to E12.5 (e–h). Low magnification views of E9.5 embryo sections (a,b); high magnification (c,d); low magnification views of E12.5 embryo sections (e,f); their high magnification (g,h). The intensity of signals for Liv2 peaked at E9.5, and the signals were localized at the cell membrane and in particles close to the cell membrane. Immunoreactivity declined gradually from E12.5. The cells showing strong immunoreactivity throughout the whole cytoplasm by self-fluorescence were hemocytes. Bars = 20 µm.

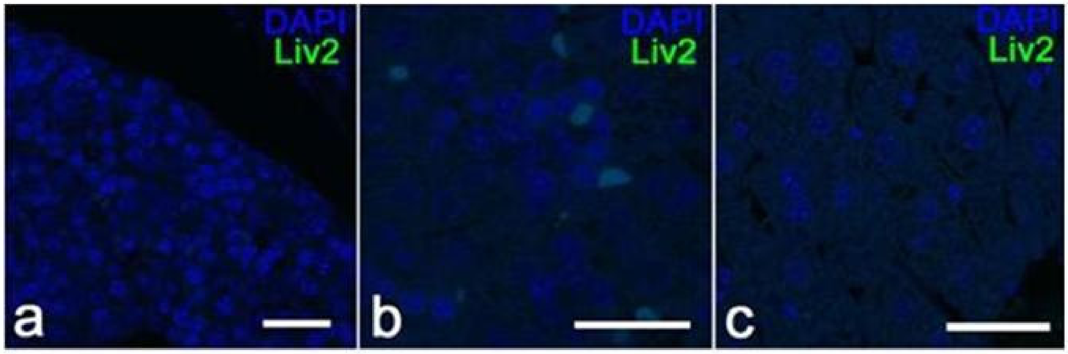

**FIGURE 3.** Liv2 (green) immunoreactivity was not observed after E14.5. Liver sections at E14.5 (a), neonate (b), and adult (c) stages. Bars = 20 µm.

## DISCUSSION

Anti-Liv2 antibody is a monoclonal antibody produced by immunization of rat lymph nodes with E11.5 embryo liver lysate and is reported to be immunoreactive only with immature hepatocytes (hepatoblasts)[14]; however, there is no information about the antigen recognized by this antibody, its estimated molecular weight, and its cell localization. We used a western blotting analysis to determine the estimated molecular weight of this antigen and its specificity.

Only one signal was found at about 40–45 kDa, and the antigen was expressed from E9.5 to E12.5. The intensity of the signal peaked at E9.5, decreased gradually thereafter, and was almost undetectable after E12.5. These observations suggest that Liv2 is specific for the early stage of hepatic development, i.e., the stage of immature hepatocytes. Hepatic development begins at E8.5 by contact between parts of the endoderm and the primordium of the heart; however, due to technical difficulties, we could not determine expression at this stage.

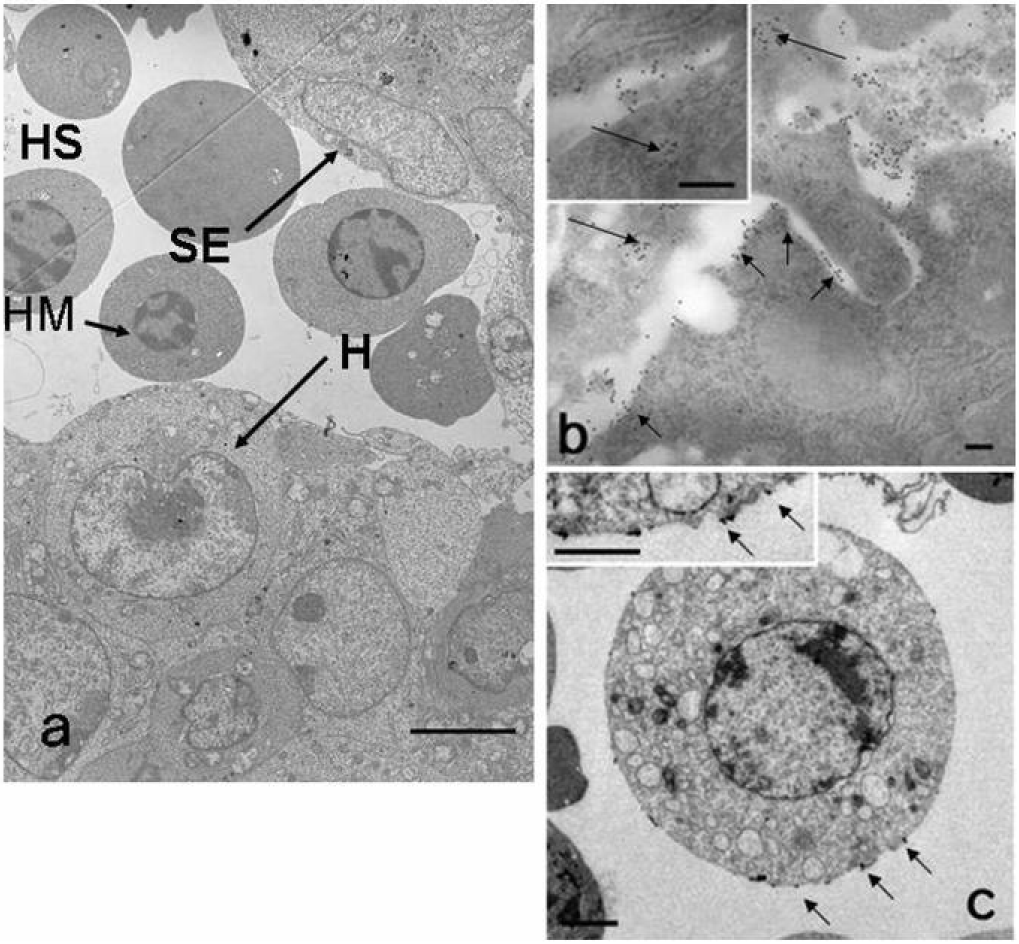

**FIGURE 4.** Ultrastructure of E11.5 immature hepatocytes. (a) TEM images of E11.5 hepatoblasts. Hemocytes were observed in the hepatic sinusoids, and endothelial cells and hepatoblasts (H) were observed underneath. (b) ImmunoGold as immuno-TEM images of E11.5 hepatoblasts immunoreactive with Liv2. The preferential distribution of gold particles for Liv2 was evident at the cell boundary and in vesicles close to the cell membrane. Inset in (b) shows higher magnification of another region. (c) Iron bead clusters (arrows) were detected on dissociated immature hepatocytes of E9.5 treated with Liv2 antibody and immunoiron beads. Inset in (c) is higher magnification of the part of the cell surface membrane to show definite iron beads (arrows). Bars = 5 µm (a); 200 nm (b); 1 µm (c).

On immunohistochemical analysis, Liv2 was shown to be localized at the cell membrane and in granules close to the cell membrane in immature hepatocytes from E9.5 to E12.5. These results are in accordance with those of the immuno-TEM analysis. It has been reported that Liv2-positive cells in the fetal liver can be separated by FACS[11]. Therefore, the epitope recognized by the anti-Liv2 antibody may be a cell surface antigen, indicating that anti-Liv2 antibody could be useful as a *de novo* marker of immature hepatocytes and for selection.

Immunocytochemical analysis was performed to verify the presence of Liv2 in immature hepatocytes derived from ES cells. Because EB outgrowth was not stimulated by specific, inductive growth factors, only a few immature hepatocytes could not be gained, but we found clusters of Liv2-positive cells in EB outgrowth with the same patterns as the immunoreactivity seen in the E9.5 immature hepatocytes. According to a previous report, hepatocytes at the earliest stage do not initially express ALB, but express it later[13]. The same findings were observed in Liv2-positive clusters. ALB was expressed in amniocytes as well, but these cells were not immunoreactive for Liv2 (data not shown). Therefore, we suggest that these Liv2-positive clusters are immature hepatocytes and that anti-Liv2 antibody can be used to detect immature hepatocytes derived from ES cells.

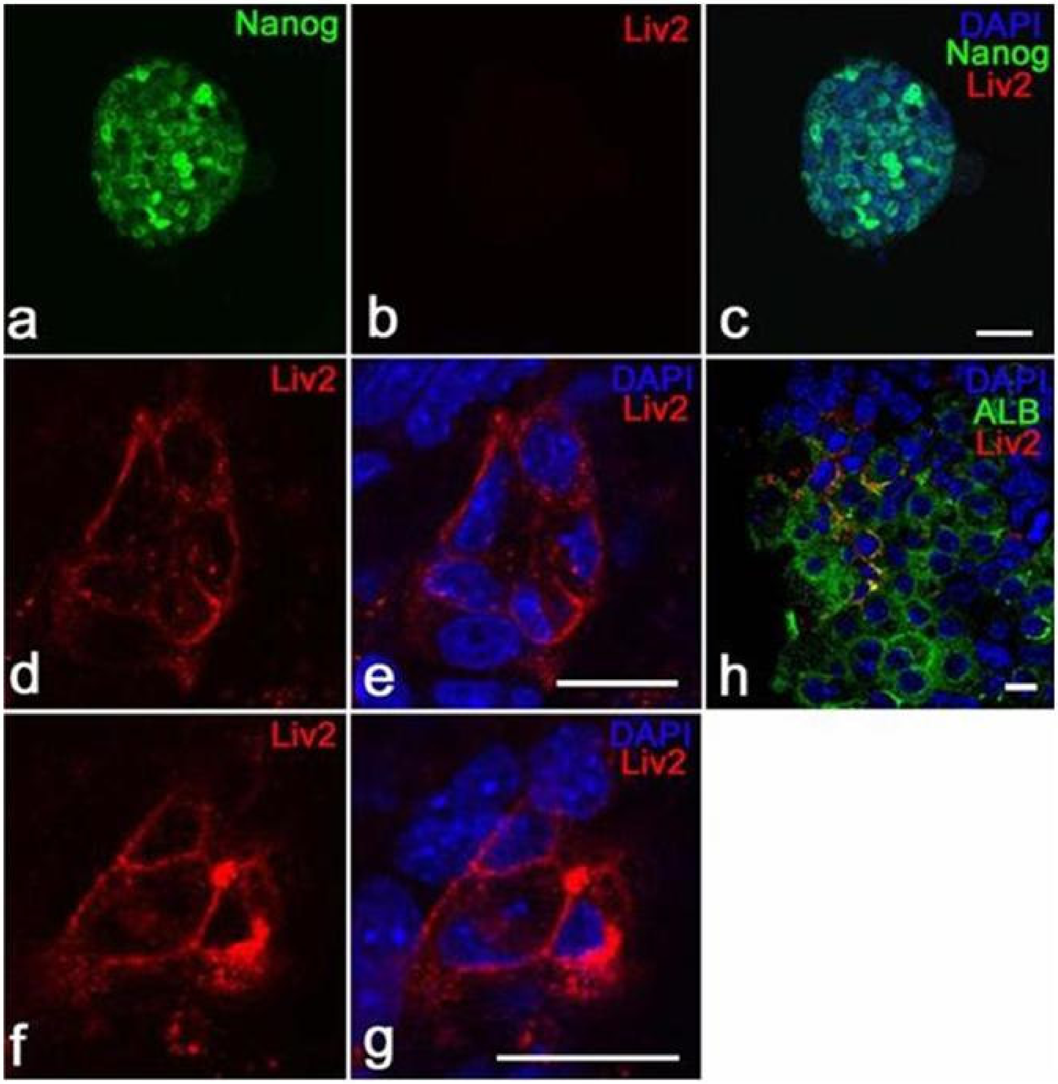

**FIGURE 5**. Liv2 immunoreactivity in ES cells (a–c) and EB outgrowth (d-h). (a–c) Liv2 (red) immunoreactivity was not seen in ES cells, which were positive for the marker of undifferentiated cells, Nanog (green). (d–g) EBs on day 7 showed Liv2-positive clusters consisting of a few cells with the same pattern of immunoreactivity as seen in fetal hepatocytes. (h) In EB outgrowth on day 18, Liv2-positive cells were merged with ALB, which is a marker of hepatocytes. Bars = 20 µm.

However, many immunopositive granules around nuclei that were different from the ones in immature hepatocytes were dispersed in the cytoplasm of epithelial cells in the peripheral region of the EB outgrowth. In addition, the signals did not merge with the nestin neuronal-positive cells or with the diffusible ALB marker of hepatocytes, even with prolonged cell culture. There are two possibilities. First is that the granules show a process of Liv2 formation, which is produced in intracellular organellae, stocked in the vesicle, and transported to the membrane, although it is unknown whether it is a transmembrane protein or absorbed protein. The second is that the anti-Liv2 antibody may immunoreact not only with immature hepatocytes, but also with other types of cells or epitopes. Molecular identification of Liv2 and an analysis of differences in immunoreactivity between immature hepatocytes and other cell types are needed before Liv2 can be used as a marker for immature hepatocytes. However, Liv2 seems to be relatively restricted to the surface of immature hepatocytes and may be useful to select immature hepatocytes derived from ES cells.

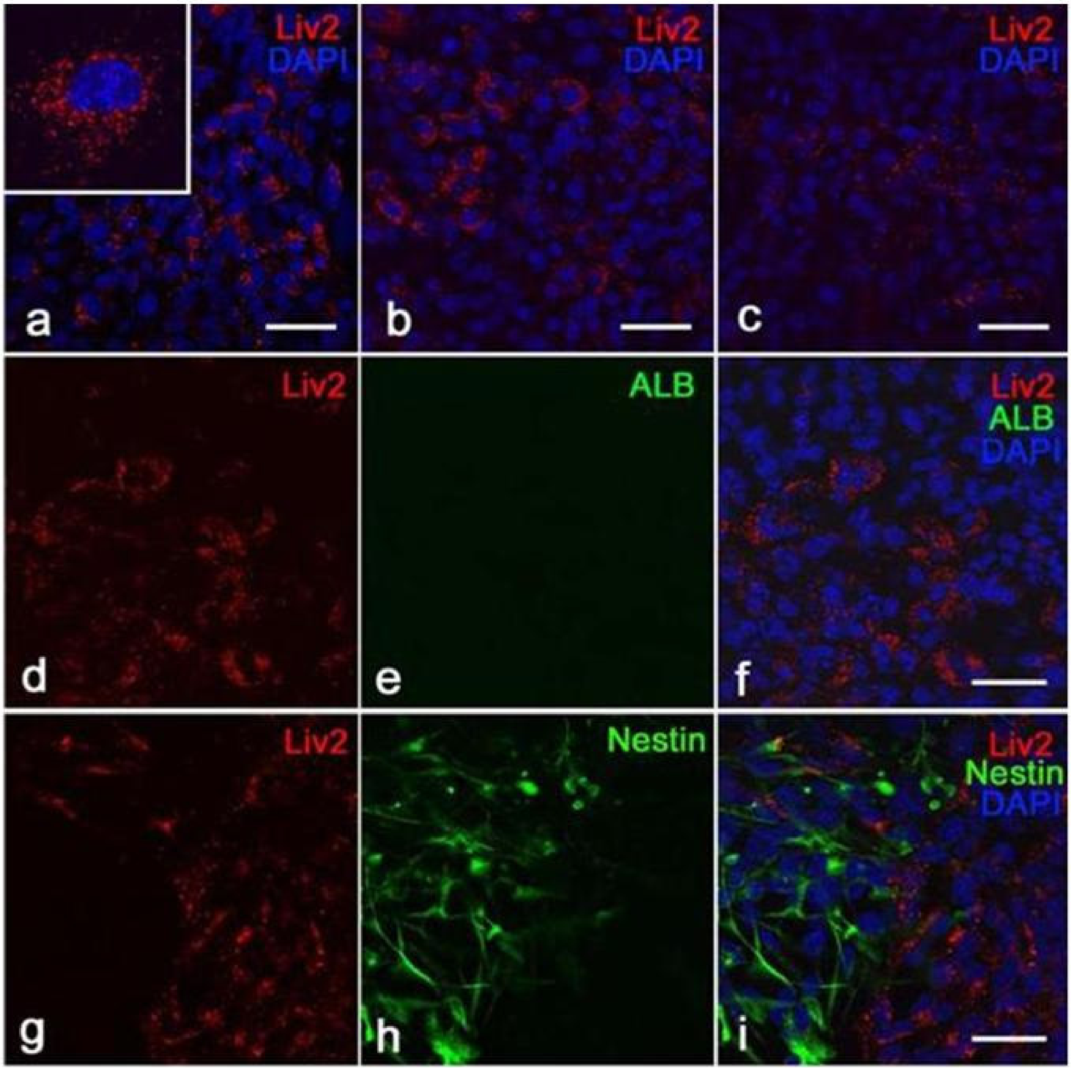

**FIGURE 6.** Another type of Liv2 chronological immunoreactivity in EB outgrowth (a, day 7; b, day 11; c, day 13) and immunocytochemical analysis of ALB and nestin (d–f, ALB, day 18; g–i, nestin, day 23). There were many granules immunoreactive for Liv2 (red) in the peripheral cells in EB outgrowth (a). Fluorescent signals were located only in the cytoplasm around the nuclei (a, inset). The number of granules decreased with prolongation of culture of EB outgrowth (b,c). The cells containing many granules did not merge with signals for ALB (f) or nestin (i). Bars = 50 μm.

## ACKNOWLEDGMENTS

We thank Ms. Kayo Suzuki and Dr. Kiyokazu Kametani (Research Center for Instrumental Analysis, Shinshu University) for their outstanding technical assistance.
